# Inactivation of PRMT5 by PARP Inhibitors Confers High Susceptibility in MTAP-Deficient Cancers

**DOI:** 10.3390/cancers18091335

**Published:** 2026-04-22

**Authors:** Qi Liu, Yuling Sheng, Folan Lin, Haoyuan Tan, Yanyun Chang, Xiaopeng Lu, Hao Chen, Changzheng Du

**Affiliations:** 1 Key University Laboratory of Metabolism and Health of Guangdong, Biochemistry Department, School of Medicine, Southern University of Science and Technology, Shenzhen 518055, China; 12231370@mail.sustech.edu.cn (Q.L.); 12131352@mail.sustech.edu.cn (Y.S.); 12333001@mail.sustech.edu.cn (H.T.); 2 Department of Pharmacology, Joint Laboratory of Guangdong & Hong Kong Universities for Vascular Homeostasis and Diseases, SUSTech Homeostatic Medicine Institute, School of Medicine, Southern University of Science and Technology, Shenzhen 518055, China; 12231355@mail.sustech.edu.cn; 3 Cancer Center, Beijing Tsinghua Changgung Hospital, School of Clinical Medicine, Tsinghua Medicine, Tsinghua University, Beijing 102218, China; cyya05042@btch.edu.cn; 4 Department of Biochemistry and Molecular Biology, Shenzhen University Medical School, Shenzhen 518055, China; luxiaopeng@szu.edu.cn; 5 Department of Human Cell Biology and Genetics, Joint Laboratory of Guangdong & Hong Kong Universities for Vascular Homeostasis and Diseases, School of Medicine, Shenzhen Key Laboratory of Gene Regulation and Systems Biology, Southern University of Science and Technology, Shenzhen 518055, China

**Keywords:** MTAP, PARP, PRMT5, cancer, therapy

## Abstract

**Background:** Methylthioadenosine phosphorylase (MTAP) deficiency caused by homozygous deletion is prevalent in solid tumors and contributes to malignant progression, thereby rendering cancer cells vulnerable to dysfunction of protein arginine methyltransferase 5 (PRMT5). This study aimed to investigate whether Poly (ADP-ribose) polymerase (PARP) inhibitors could exploit this vulnerability through PRMT5 inactivation in MTAP-deficient tumors. **Methods:** PRMT5 activity was assessed in vitro and in vivo following PARP inhibitor treatment. The antitumor effects of PARP inhibitors alone or in combination with either the MTAP inhibitor MTDIA or the PRMT5 inhibitor EPZ015666 were evaluated in solid tumor models, including MTAP-deficient tumor models in vivo. **Results:** PARP inhibitors effectively inactivated PRMT5 in vitro and in vivo and exacerbated DNA double-strand breaks induced by PARP inhibition. Moreover, PARP inhibitors showed significant synergistic effects when combined with either MTDIA or EPZ015666 in solid tumor models. MTAP-deficient tumors exhibited increased vulnerability to olaparib in vivo, and combined treatment with olaparib plus MTDIA or EPZ015666 produced improved therapeutic outcomes compared with olaparib alone. **Conclusions:** These findings identify PARP inhibitors as a potential therapeutic strategy for MTAP-deficient tumors through targeted inactivation of PRMT5 and support further evaluation of PARP inhibitor-based combination therapies in this molecular context.

## Simple Summary

MTAP deficiency is a common genetic alteration in solid tumors and may create a therapeutic vulnerability. In this study, we investigated whether PARP inhibitors could provide a treatment advantage in MTAP-deficient cancers. We found that PARP inhibitors not only impair DNA damage repair, but also suppress PRMT5 activity, a function on which MTAP-deficient cancer cells are particularly dependent. Consistent with this, MTAP-deficient tumors showed increased sensitivity to PARP inhibition in both cell-based and animal models. In addition, the combination of PARP inhibitors with agents targeting MTAP or PRMT5 produced stronger antitumor effects than PARP inhibition alone. These findings indicate that PARP inhibitor-based strategies may represent a useful therapeutic approach for MTAP-deficient cancers and support further evaluation of such combination treatments.

## 1. Introduction

MTAP deficiency resulting from homozygous deletion of the chromosomal region 9p21.3 is prevalent across various cancer types [1], particularly in pleural mesotheliomas, malignant glioma, urothelial carcinoma and pancreatic adenocarcinoma [2]. MTAP is a key enzyme in the salvage synthesis pathway of adenine and methionine, catalyzing the phosphorylation and cleavage of S-methyl-5′-thioadenosine (MTA) into adenine and 5-methylthioribose-1-phosphate (MTR), which is subsequently converted into methionine [3]. Methionine could be utilized to synthesize S-adenosylmethionine (SAM), the major methyl donor in biochemical processes and substrate for the protein arginine N-methyltransferase 5 (PRMT5) [4], a type II arginine methyltransferase involved in numerous cancer-associated regulatory pathways, including cell proliferation and differentiation, DNA repair, apoptosis and immune evasion [5,6,7]. The loss of MTAP leads to an accumulation of MTA that partially inactivates PRMT5 [1], rendering MTAP-deficient tumors heavily reliant on residual PRMT5 activity; this dependency provides an opportunity for therapeutic intervention based on synthetic lethality [1,8].

Poly (ADP-ribose) polymerases (PARPs) comprise a family of 17 proteins involved in the DNA damage repair, stress response and apoptosis [9]. PARP inhibitors (PARPis) are a class of anticancer agents approved for treating homologous recombination (HR) repair-deficient tumors such as BRCA1/2 mutated breast, ovarian, and pancreatic cancers [10]. In this study, we reveal that PARPis directly inhibit PRMT5 in vitro and in vivo, rendering the MTAP-deficient tumors highly vulnerable to PARPi treatment. This discovery offers a novel strategy for the treatment of MTAP-deficient cancers.

## 2. Results

### 2.1. PARP Inhibitor Olaparib Inactivates PRMT5 In Vitro and In Vivo

**Figure 1:**
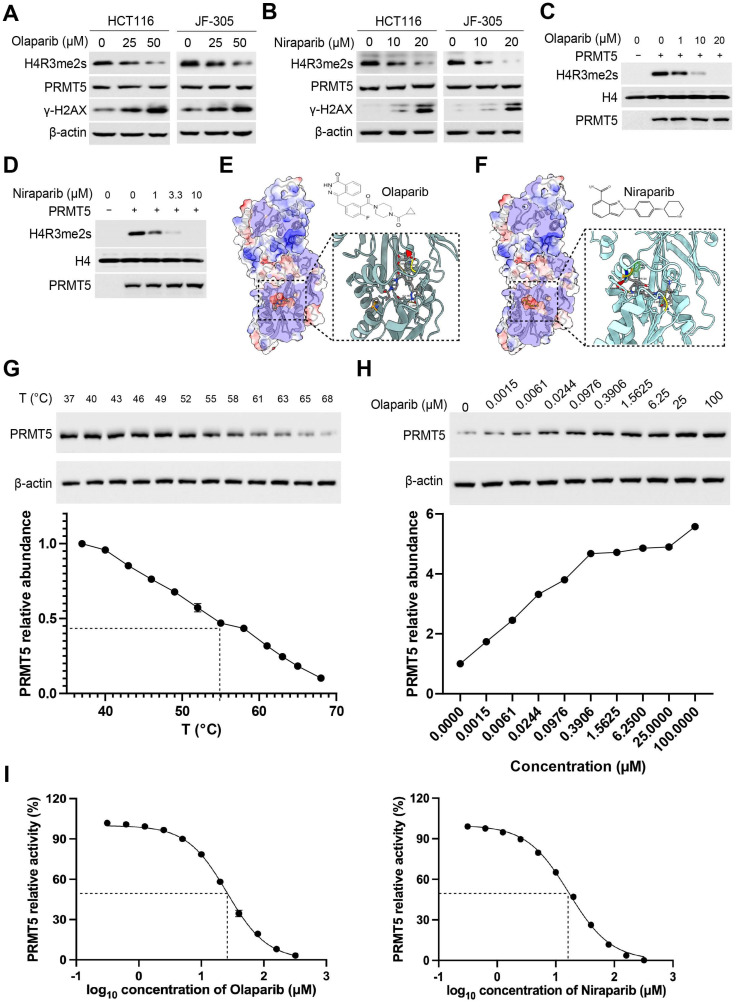
PARP inhibitors inactivate PRMT5. (**A**,**B**) PARP inhibitors olaparib (**A**) and niraparib (**B**) induce demethylation of H4R3 in HCT116 and JF-305 cells, without change in PRMT5 protein abundance, determined by immunoblotting. (**C**,**D**) In vitro assay detecting PRMT5 activity following treatment of olaparib (**C**) or niraparib (**D**), determined by immunoblotting. (**E**,**F**) Simulation-based docking model depicts the interaction of PRMT5 with olaparib (**E**) or niraparib (**F**). (**G**) Thermal stability of PRMT5 determined by CETSA immunoblotting. (**H**) Immunoblots of dose-dependent CETSA of olaparib-PRMT5 incubated at 55 °C. (**I**) Dose–response curves of olaparib and niraparib on PRMT5 enzymatic activity in vitro, showing IC_50_ values of approximately 25 μM and 20 μM, respectively. Original full-length WB images corresponding to this figure are provided in Figure S3.

When investigating histone arginine methylation modification, we observed an interesting phenomenon that PARPi, such as olaparib and niraparib, induce a reduction in H4R3 dimethylation (H4R3me2s) (Figure 1A,B), without affection on the protein abundance of the arginine methyltransferase PRMT5. The in vitro enzymatic assays revealed that both olaparib and niraparib significantly inhibit PRMT5 activity (Figure 1C,D). To gain a deep insight into the underlying mechanism, we performed a docking analysis of PARPi with PRMT5. The results demonstrated that both olaparib and niraparib exhibit high affinity for the SAM-dependent methyltransferase domain of PRMT5 (Figure 1E,F, Tables S1 and S2), responsible for its catalytic activity [11]. To further elucidate the interaction between PARPi and PRMT5, we conducted a cellular thermal shift assay (CETSA) combined with immunoblotting to evaluate olaparib-PRMT5 binding (Figure 1G,H). The CETSA results indicated that olaparib enhances the thermal stability of PRMT5, suggesting a direct interaction between PARPi and PRMT5. Using the MTase-Glo methyltransferase assay, the apparent IC_50_ values were approximately 25 μM for olaparib and 20 μM for niraparib (Figure 1I). In addition, to eliminate the potential influence of PARPi on upstream proteins of PRMT5, such as MAT2A, as previously reported in breast cancer studies [12], we assessed the mRNA levels of MAT2A. Our findings demonstrated that there is no significant change in its expression following PARPi treatment (Figure S1). The original full-length WB images corresponding to Figure 1 are provided in Figure S3.

### 2.2. PRMT5-Knockdown or MTAP-Knockout Cancer Cells Have a High Vulnerability to PARPi

**Figure 2:**
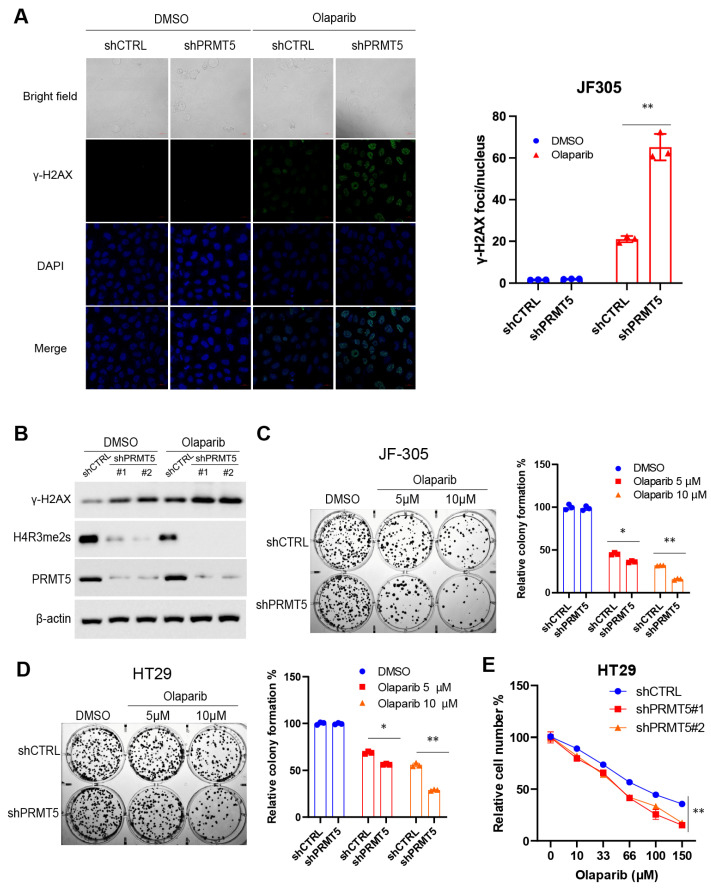
*PRMT5* knockdown cells are highly vulnerable to PARP inhibitors. (**A**) γH2AX foci formation induced by Olaparib in JF-305 derivative cell lines (shCTRL or shPRMT5), determined by immunofluorescence assay. (**B**) The abundance of γH2AX following Olaparib treatment in JF-305 derivative cells (shCTRL or shPRMT5), determined by immunoblotting. (**C**,**D**) JF-305 (**C**) or HT29 (**D**) derivative cell lines (shCTRL or shPRMT5) were treated with Olaparib for 24 h, and colony formation assays were performed with 500 per well to determine the proliferation ability of treated cells. (**E**) HT29 derivative cell lines (shCTRL or shPRMT5) were treated with Olaparib for 48 h, and cell numbers were determined by CCK8 assay. Original full-length WB images corresponding to this figure are provided in Figure S4. * *p* < 0.05; ** *p* < 0.01.

To illuminate the pharmacologic significance of the inhibitory effect of PARPi on PRMT5, we treated cancer cells with *PRMT5* knockdown and their control counterparts using PARPi. Our findings revealed that PARPi exacerbates DNA double-strand breaks (DSB) in *PRMT5* knockdown cells, as evidenced by an increased level of γ-H2AX detected through immunofluorescence (Figure 2A) and immunoblotting assays (Figure 2B). Correspondingly, the cytotoxic effect of PARPi is significantly higher in *PRMT5* knockdown cells compared to their control counterparts, as determined by colony formation (Figure 2C,D) or CCK8 assay (Figure 2E). The original full-length WB images corresponding to Figure 2 are provided in Figure S4.

**Figure 3:**
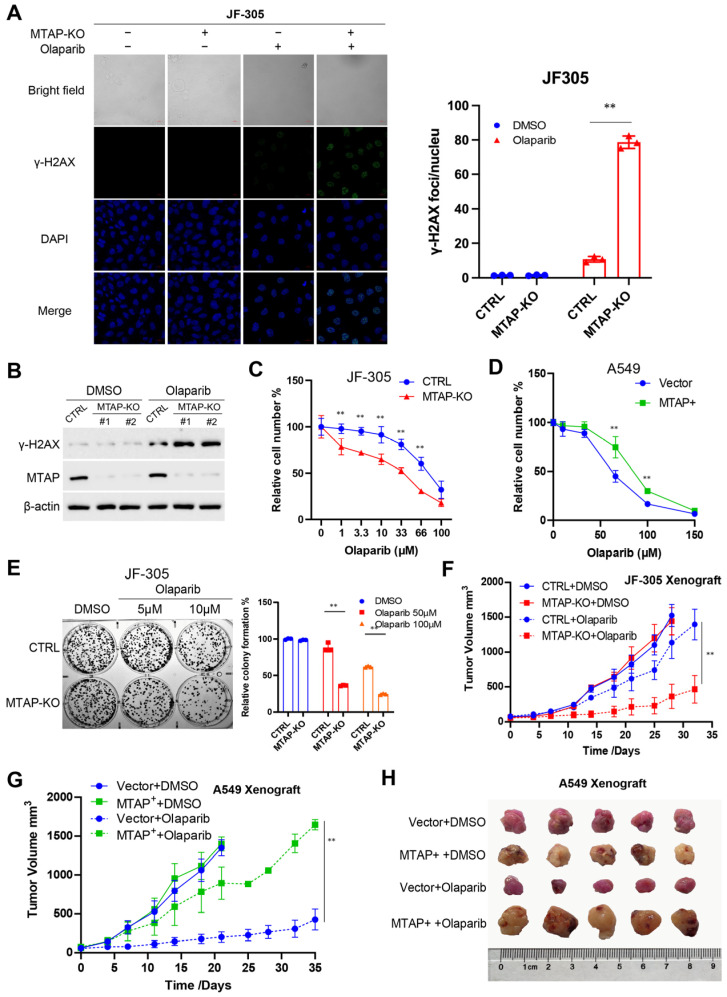
MTAP knockout cells are highly vulnerable to PARP inhibitors. (**A**) γH2AX foci formation induced by Olaparib in JF-305 derivative cell lines (CTRL or *MTAP*-KO), determined by immunofluorescence assay. (**B**) The abundance of γH2AX following Olaparib treatment in JF-305 derivative cells (CTRL or *MTAP*-KO), determined by immunoblotting. (**C**,**D**) JF-305 derivative cell lines (CTRL or *MTAP*-KO) (**C**) or A549 derivative cell lines (Vector or *MTAP*+) (**D**) were treated with Olaparib for 48 h, and cell numbers were determined by CCK8 assay. (**E**) JF-305 derivative cell lines (CTRL or *MTAP*-KO) were treated with Olaparib for 24 h, and colony formation assays were performed with 500 per well to determine the proliferation ability of treated cells. (**F**–**H**) Growth curve of subcutaneous xenograft tumors of JF-305 derivative cells (CTRL or *MTAP*-KO) (**F**) or A549 derivative cells (Vector or *MTAP*+) (**G**) treated by olaparib or vehicle (PBS); and tumor size of A549 xenografts in harvest (**H**). Original full-length WB images corresponding to this figure are provided in Figure S5. ** *p* < 0.01.

Given that MTAP-deficient tumors are heavily reliant on residual PRMT5 activity [13], we hypothesized that this tumor genotype may exhibit heightened susceptibility to PARPi. To validate this hypothesis, we knocked out MTAP in JF-305 cells and treated the MTAP-KO cells and their control counterparts with PARPi. We observed that olaparib induces more severe DSB in MTAP-KO cells compared to their control counterparts (Figure 3A,B). Furthermore, the cytotoxicity of PARPi was markedly enhanced against MTAP-deficient cancer cells, as demonstrated by CCK8 assays (Figure 3C,D) and colony formation assays (Figure 3E). To evaluate the therapeutic efficacy of PARPi in vivo, we established a subcutaneous xenograft model with either MTAP-KO or MTAP-normal JF-305 cells. Mice were treated with PARPi to simulate clinical cancer treatment scenarios. The results demonstrated that MTAP-KO group exhibited superior therapeutic outcomes, characterized by the most significant inhibition of tumor growth (Figure 3F). In contrast, restoring MTAP expression in A549 cells (Figure S2), a lung cancer cell line deficient in MTAP, resulted in notable resistance to PARPi in a xenograft tumor model (Figure 3G,H). Collectively, these findings suggest that cancers characterized by either *PRMT5* knockdown or MTAP deficiency display heightened vulnerability to treatment with PARP inhibitors. The original full-length WB images corresponding to Figure 3 and Supplementary Figure S2 are provided in Figure S5 and Figure S6, respectively.

### 2.3. Olaparib Has Synergistic Effect with PRMT5 or MTAP Inhibitor

**Figure 4:**
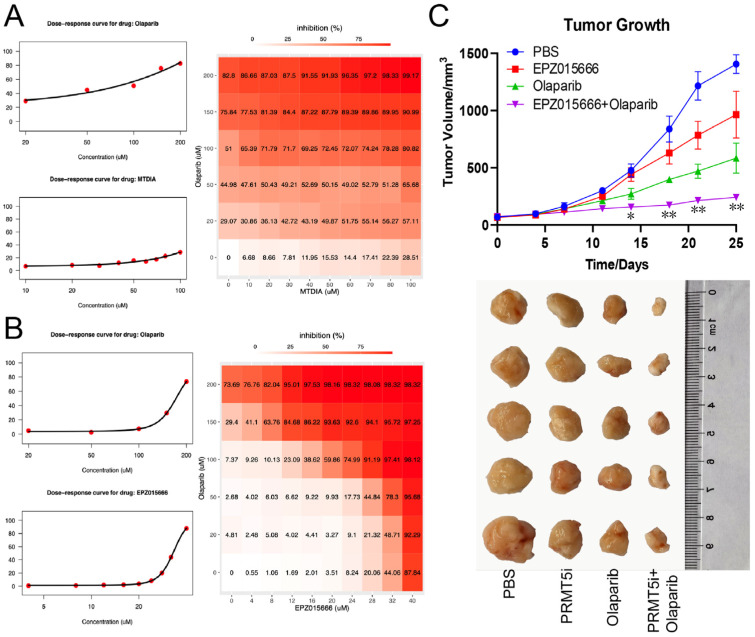
Synergistic effect of PARP inhibitors and the inhibitors of MTAP or PRMT5. (**A**,**B**) Synergistic effect of olaparib and MTDIA (**A**) or EPZ015666 (**B**) by SynergyFinder 2.0 model (ZIP method). The synergistic effect of olaparib and MTDIA or EPZ015666 were determined to be significant (*p* < 0.01). (**C**) Growth curve of subcutaneous xenograft tumors of JF-305 cells treated by olaparib or vehicle (PBS), without or with PRMT5 inhibitor EPZ015666 (upper panel); and tumor size in harvest (bottom panel). * *p* < 0.05; ** *p* < 0.01.

Based on the synthetic lethality observed with PARPi in conjunction with *MTAP/PRMT5* deficiency, we further explored the synergistic effects of PARPi combined with either MTAP or PRMT5 inhibitors. First, we treated JF-305 cells with olaparib and MTDIA, a commonly utilized MTAP inhibitor. The results suggested a significant synergistic effect in inducing cancer cell death (ZIP score 14.591, *p* < 0.01) (Figure 4A). Second, HCT116 cells were treated with olaparib and EPZ015666, a PRMT5 inhibitor; this combination also demonstrated a notable synergistic effect (ZIP score 20.708, *p* < 0.01) (Figure 4B). Finally, we administered olaparib and EPZ015666 to nude mice bearing xenograft tumors derived from HCT116 cells. We observed that the combination of olaparib and EPZ015666 yielded the most favorable therapeutic outcome, achieving maximal tumor inhibitory effects (Figure 4C).

**Figure 5:**
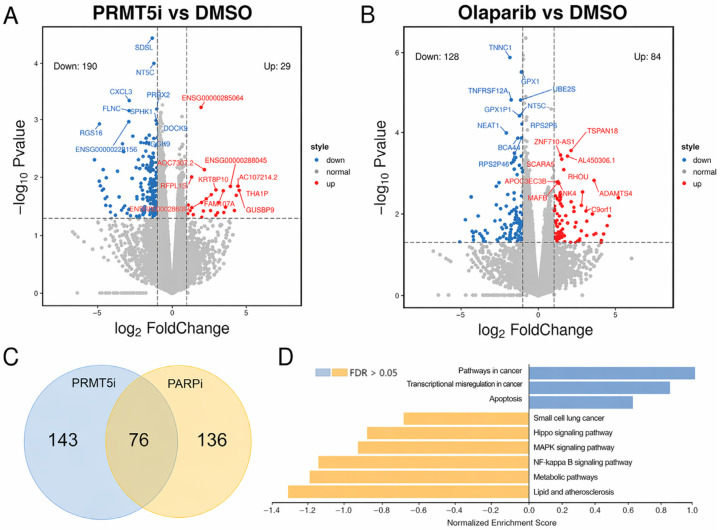
The alteration of gene expression following treatment with PRMT5 or PARP inhibitors. (**A**,**B**) Volcano blotting of differentially expressed gene (DEGs) following treatment of PRMT5 inhibitor (EPZ015666) (**A**) or PARP inhibitor (olaparib) (**B**), determined by RNA-seq. (**C**) The overlap DEGs between PRMT5i and PARPi groups. (**D**) KEGG pathway enrichment analysis of the overlap genes between PRMT5i and PARPi groups.

To deeply understand the synergistic effect between PARPi and PRMT5i, we investigated changes in gene expression following treatment with either PARPi or PRMT5i (Figure 5). The number of differentially expressed genes (DEGs) identified in the PRMT5i and PARPi groups were 219 and 212, respectively (Figure 5A,B), with an overlap of 76 DEGs between the two groups (Figure 5C). KEGG pathway enrichment analysis for these 76 genes indicated their involvement in significant cancer-associated signaling pathways, including transcriptional misregulation, apoptosis, MAPK pathway, NF-κB pathway, among others.

## 3. Materials and Methods

### 3.1. Cell Lines and Cell Culture

HT-29, HCT116 and HEK-293T cells were purchased from Pricella Biotechnology, Co., Ltd. (Wuhan, China) which were obtained from the American Type Culture Collection (ATCC) or European Collection of Authenticated Cell Cultures (ECACC). JF-305 cells were kindly provided by Prof. Jing Gao, and A549 cells were a gift from Prof. Guoan Chen. Cell line identity was verified through short tandem repeat (STR) profiling. HT-29, JF-305, and A549 cells were maintained in RPMI-1640 medium (Gibco, Thermo Fisher Scientific, Waltham, MA, USA, Cat#61870036) containing 10% fetal bovine serum (FBS; Gibco, Thermo Fisher Scientific, Waltham, MA, USA, Cat#10099141C) and 1% penicillin–streptomycin (Gibco, Thermo Fisher Scientific, Waltham, MA, USA, Cat#15140122). In contrast, HCT116 and HEK293T cells were grown in Dulbecco’s Modified Eagle Medium (DMEM; Gibco, Thermo Fisher Scientific, Waltham, MA, USA, Cat#11995065). All cultures were kept in a humidified incubator at 37 °C with 5% CO_2_. Each cell line was used within 20 passages and was routinely screened to exclude mycoplasma contamination.

### 3.2. Plasmids, Chemicals and Antibodies

A complete list of reagents, including chemicals and antibodies, is provided in Table S3. Stable knockdown of *PRMT5* was achieved by introducing two shRNAs into the pLKO.1-TRC vector (Addgene, Watertown, MA, USA, #10878). For *MTAP* editing, lentiCRISPR v2 (Addgene, Watertown, MA, USA, #52961) containing two sgRNAs was used to generate knockout clones in JF-305 cells, while *MTAP* overexpression in A549 cells was established with the pLV [Exp]-EGFP/Puro-EF1A > mCherry construct (VectorBuilder, Guangzhou, China). Correct insertion of all plasmids was validated through Sanger sequencing. Lentiviral particles were produced in HEK293T cells (ATCC, Manassas, VA, USA, Cat#CRL-3216), and target cells were infected followed by selection using 1 µg/mL puromycin (MedChemExpress, Monmouth Junction, NJ, USA, #HY-K1057). The primers applied in plasmid assembly are listed in Table S4.

### 3.3. Cell Proliferation and Viability Assays

Cell viability and proliferation were measured using the Cell Counting Kit-8 (Beyotime, Shanghai, China, Cat#C0040). Approximately 1 × 10^3^ cells were plated in each well of a 96-well plate and cultured for 24 h prior to treatment with different concentrations of the indicated compounds. Subsequently, 10% CCK-8 reagent was added to each well and incubated at 37 °C for 1–4 h. The absorbance at 450 nm was recorded using a Synergy HTX microplate reader (BioTek Instruments, Winooski, VT, USA). Cell viability was expressed relative to untreated controls, which were set as 100%. GraphPad Prism v10 (GraphPad Software, San Diego, CA, USA) was employed to generate dose–response curves using its built-in simulation function. Each dose was tested in quintuplicate, and three independent experiments were performed.

To further evaluate proliferative capacity, a colony formation assay was conducted. 500 cells were seeded per well in 6-well plates with fresh medium and cultured for 12–18 days. Colonies containing ≥50 cells were fixed with 4% paraformaldehyde and stained with 0.1% crystal violet solution. Colonies were photographed and quantified using ImageJ software (v1.54, NIH, USA). Plating efficiency (PE) was calculated as the number of colonies divided by the number of seeded cells, expressed as a percentage. For drug-treated samples, the surviving fraction (SF) was calculated by normalizing the colony formation rate to the PE of untreated controls. All experiments were carried out in triplicate, and significance testing was performed using Student’s *t*-test.

### 3.4. RNA Extraction, Reverse Transcription and Quantitative Real-Time PCR (RT-qPCR)

Total RNA was isolated from cultured cells using the RNA extraction kit (Vazyme, Nanjing, China, Cat# RC112-01) according to the manufacturer’s instructions. One microgram of RNA was reverse-transcribed into complementary DNA (cDNA) using the reverse transcription kit (Vazyme, Nanjing, China, Cat# R312-02). Quantitative PCR was then performed on a QuantStudio 7 Flex instrument (Life Technologies, Carlsbad, CA, USA) using the SYBR Green PCR kit (TransGen, Beijing, China, Cat# AQ132-21). Primer sequences are provided in Table S4. Gene expression was normalized to *B2M* as the internal control. All experiments were conducted in triplicate.

### 3.5. Protein Extraction and Immunoblots

Whole-cell lysates were generated using RIPA buffer supplemented with protease inhibitor cocktail (Beyotime, Shanghai, China, Cat# P1005). Following PBS washes, cells were lysed and the extracts were heated to 100 °C for 10 min. Protein levels were quantified with the BCA assay kit (Thermo Fisher Scientific, Waltham, MA, USA, Cat# 23227). Approximately 40–50 µg of protein per lane was loaded onto 8–12% SDS-PAGE gels for electrophoretic separation and subsequently transferred onto PVDF membranes (Millipore, Burlington, MA, USA, Cat# IPFL00010). The membranes were blocked in 5% skim milk for 1 h at room temperature, then probed with primary antibodies overnight at 4 °C. After incubation with HRP-linked secondary antibodies (ZSGB-Bio, Beijing, China, Cat# ZB-2301, ZB-2305) for 1 h, protein bands were detected by enhanced chemiluminescence (Advansta, San Jose, CA, USA, Cat# K-12045-D50). Images were acquired with a ChampChemi 610 Plus imaging platform (Sage Creation, Beijing, China), and band intensity was quantified using ImageJ software (v1.54, NIH, Bethesda, MD, USA). Details of antibody information are provided in Table S3.

### 3.6. Immunofluorescent Staining

Cells were plated on µ-Slide 8-well chambers (ibidi, Gräfelfing, Germany, Cat#80806) and cultured until they reached 60–70% confluence. Cultures were then exposed to either PARP inhibitor or vehicle (DMSO) for 24 h in a humidified incubator. Following treatment, cells were washed with PBS and fixed for 1 h in 4% paraformaldehyde. Permeabilization was achieved by incubation with 0.25% Triton X-100 (Thermo Fisher Scientific, Waltham, MA, USA, Cat#85111) for 10 min. Non-specific binding was blocked with 5% BSA for 1 h, after which cells were incubated overnight at 4 °C with an antibody against γ-H2AX (Abcam, Cambridge, UK, Cat# Ab26350). After washing, fluorescently labeled secondary antibody (HUABIO, Hangzhou, China, Cat# HA1112) was applied for 1 h in the dark. Nuclei were counterstained with DAPI (Beyotime, Shanghai, China, Cat# C1341S) for 10 min, and samples were maintained in PBS (150 µL per well). Fluorescence images were collected on a Zeiss LSM 980 confocal microscope (Carl Zeiss, Oberkochen, Germany), and γ-H2AX foci were analyzed with ImageJ software (v1.54, NIH Bethesda, MD, USA).

### 3.7. Mouse Xenografts and Treatments

NCG male mice aged 4–6 weeks were purchased from the Shanghai Model Organisms Center (Shanghai, China). Mice were maintained in a specific pathogen-free (SPF) facility, and all procedures were approved by the Animal Ethics Committee of the Southern University of Science and Technology. To establish subcutaneous xenografts, 3 × 10^6^ tumor cells were injected into the dorsal flank region. Body weight and tumor volume were recorded twice per week. Tumor volume was calculated using the formula: V = (length × width^2^)/2. When tumor size reached approximately 100 mm^3^, animals were randomly allocated to one control group and three treatment groups. Treatments consisted of intraperitoneal administration of the test compounds formulated in corn oil, given every 3 days for a total of 3 weeks. Mice were euthanized by CO_2_ exposure followed by cervical dislocation if tumors grew beyond 15 mm in diameter or 1500 mm^3^ in volume. Tumors were removed post-mortem and photographed.

### 3.8. In Vitro Methylation Assay

For in vitro methylation assays, 0.5 µg of purified histone H4 protein (Abcam, Cat#198115) was combined with 0.3 µg of recombinant PRMT5 enzyme (Active Motif, Carlsbad, CA, USA, Cat#31393). Reactions (30 µL) were assembled in buffer containing 50 mM Tris-HCl (pH 8.6), 0.02% Triton X-100, 2 mM MgCl_2_, 1 mM TCEP, and 50 µM S-adenosyl-L-methionine (SAM). Mixtures were incubated for 3 h at room temperature under five specified conditions. Reactions were terminated by addition of 2× SDS sample buffer, followed by heat denaturation at 100 °C for 10 min. 6 µL of each reaction were resolved on 12% SDS-PAGE gels and analyzed by immunoblotting with antibodies against H4R3me2s (Abcam, Cambridge, UK, Cat#5823), PRMT5 (Cell Signaling Technology, Danvers, MA, USA, Cat#79998S), and H4 (Cell Signaling Technology, Danvers, MA, USA, Cat#2935T).

PRMT5 methyltransferase activity was measured using the MTase-Glo™ Methyltransferase Assay (Promega, Madison, WI, USA), which detects the reaction product S-adenosylhomocysteine (SAH) through a luminescence-based readout. Each 20 µL reaction contained 0.1 µg recombinant PRMT5, 0.1 µg histone H4, 10 µM S-adenosyl-L-methionine (SAM), and olaparib/niraparib at the indicated concentrations in 1× reaction buffer. Olaparib was tested using a serial dilution gradient, while vehicle control wells received an equal volume of DMSO; the final DMSO concentration was maintained at 1% in all wells. Reactions were incubated for 3 h at room temperature, followed by addition of 5 µL 5× MTase-Glo™ Reagent and 25 µL MTase-Glo™ Detection Solution according to the manufacturer’s instructions. Luminescence was measured on a GloMax^®^ Navigator Microplate Luminometer (Promega, Madison, WI, USA). Background-subtracted signals were normalized to the vehicle control, and apparent IC_50_ values were determined by nonlinear regression in GraphPad Prism 10 (GraphPad Software, San Diego, CA, USA). Each condition was assayed in triplicate.

### 3.9. Cellular Thermal Shift Assay (CETSA)

CETSA was performed by first washing cells twice with ice-cold PBS and harvesting them by scraping. Cell pellets were lysed in buffer containing 50 mM HEPES (pH 7.5), 5 mM β-glycerophosphate, 0.1 mM activated Na_3_VO_4_, 10 mM MgCl_2_, and 1 mM TCEP, supplemented with freshly added EDTA-free protease inhibitor cocktail (Beyotime, Shanghai, China, Cat# P1005). Lysis was facilitated by three freeze–thaw cycles alternating liquid nitrogen and a 37 °C water bath for 10 min each, followed by 10 passages through a pipette tip for mechanical disruption. The lysates were centrifuged at 21,000× *g* for 20 min at 4 °C, and the supernatants were collected. Protein concentrations were determined using the BCA Protein Assay Kit (Thermo Fisher Scientific, Waltham, MA, USA, Cat# 23227).

For thermal gradient CETSA, JF-305 cell lysates (100 µg of total protein per reaction) were preincubated with either 10 µM Olaparib (MedChemExpress, Monmouth Junction, NJ, USA, Cat# HY-10162) or DMSO (Thermo Fisher Scientific, Waltham, MA, USA, Cat# D12345) for 10 min at room temperature. Aliquots (30 µL) were heated in a PCR instrument for 3 min at 12 different temperatures ranging from 37 to 68 °C, and then rapidly cooled to 4 °C. Reactions were terminated by adding 2× SDS loading buffer (Beyotime, Shanghai, China, Cat# P0015).

For isothermal dose–response (ITDR) CETSA, 55 °C was selected as the optimal assay temperature based on the thermal gradient results. JF-305 lysates (100 µg protein per sample) were incubated with Olaparib across 10 concentrations prepared by 4-fold serial dilutions starting from 100 µM. Following a 10 min incubation, samples were heated at 55 °C for 3 min in a PCR cycler, cooled on ice, and then boiled at 100 °C for 10 min. All samples were subsequently analyzed by Western blotting to assess PRMT5 protein levels using specific antibodies (Cell Signaling Technology, Danvers, MA, USA, Cat# 79998S).

### 3.10. Synergistic Effect Analysis

Drug combination assays were conducted using a dose–response matrix design. Olaparib, EPZ015666 (MedChemExpress, Monmouth Junction, NJ, USA, Cat#HY-12727), and MTDIA (MCE, Cat#HY-101496) were tested individually and in pairwise combinations (Olaparib + EPZ015666, Olaparib + MTDIA) across serial dilutions. After drug treatment, cell viability was measured by CCK-8 assay, and data were analyzed using the SynergyFinder Plus platform (https://synergyfinder.org) [14]. Single-agent dose–response curves were fitted to calculate expected additive effects. Combination effects were evaluated using multiple reference models, including Bliss independence, Loewe additivity, highest single agent (HSA), and zero interaction potency (ZIP). Synergy scores were generated to quantify synergistic interactions. Z scores ≥ 10 was considered statistically significant.

### 3.11. Molecular Docking

Molecular docking simulation was performed using AutoDock Tools v1.5.7. The crystal structure of PRMT5 (PDB ID: 7MXA) served as the receptor. Ligand structures of Olaparib and Niraparib were obtained from the PubChem database. Both the receptor and pre-optimized ligand were converted to the required pdbqt format. A docking grid was defined as a 15 Å cube centered at coordinates (x = −32.711, y = 30.678, z = −4.597), and 50 binding poses were generated. The resulting poses were ranked by binding free energy, with the lowest energy conformation selected for subsequent analysis. PyMOL v3.1 (Schrödinger, New York, NY, USA) and ChimeraX v1.5 (University of California, San Francisco, CA, USA) were used for all molecular visualization tasks.

### 3.12. RNA Sequencing and Analysis

Total RNA was extracted from cultured cells for the construction of libraries using the Fast RNA-seq Lib Prep Kit V2 for Illumina (ABclonal, Wuhan, China, #RK20306) according to manufacturer’s instructions. The sequencing process was performed on the Illumina NovaSeq xplus platform (Illumina, San Diego, CA, USA) with an average of 60 million total reads per sample for RNA-seq by Haplox Biotechnology Co., Ltd. (Shenzhen, China). The raw read data underwent quality control and preprocessing by Fastp (v0.23.2), followed by mapping to the human genome through HISAT2 software (v2.2.0). Differential expression analysis of two conditions/groups (three biological replicates percondition) was performed using the DESeq R package (v1.18.1). Genes with |log2(FoldChange)| > 1 and adjusted *p* value < 0.05 found by DESeq were assigned as differentially expressed. The functional enrichment analysis was performed by the clusterProfiler software package v4.8.2, which incorporates the functional annotations from Kyoto Encyclopedia of Genes and Genomes (KEGG) and Reactome databases. *p* value < 0.05 was considered significantly enriched by differential expressed genes. Gene Set Enrichment Analysis (GSEA) v4.3.2 was used to analyze the signaling pathways enriched based on the DEGs.

### 3.13. Statistical Analysis

All data are expressed as mean ± standard deviation (SD). Differences between two groups were evaluated using a two-tailed Student’s *t*-test, whereas one-way ANOVA was applied for comparisons involving more than two groups. Statistical analyses were carried out with GraphPad Prism v10 (GraphPad Software, San Diego, CA, USA) and SPSS v24.0 (IBM, Armonk, NY, USA). A *p* value < 0.05 (two-tailed) was considered to indicate statistical significance.

## 4. Discussion

PRMT5 plays a complex role in cancer, involved in promoting numerous oncogenic processes including proliferation, migration, immune evasion and DNA damage repair via different pathways [7]; within which, the essential function of PRMT5 in accelerating DNA repair and anti-apoptosis have been well established [15]. As a major type II PRMT, PRMT5 catalyzes symmetric dimethylation of arginine residues in histone N-terminal tails, activating the expression of DNA repair genes such as RNF168 and FANCD2 [6,16]; on the other hand, PRMT5 methylates and activates DNA repair proteins such as TIP60 and TP53, to enhance the repair process and maintain the genome stability [17,18]. Therefore, inhibition of PRMT5 sensitizes cancer cells to DNA damage agents. In this context, our findings fit well with growing evidence that PRMT5 is an important regulator of homologous recombination and non-homologous end joining, and that PRMT5 inhibition reshapes DNA damage response programs in cancer cells [19,20]. Interestingly, we show that the typical DNA breaking agents PARPi could inhibit PRMT5, leading to severe DNA damage in MTAP-deficient tumor cells. This finding not only reveals a novel pharmacological effect of PARPi, but also provides a significant clue for the clinical application of PARPi. Because the SAM-dependent catalytic region is structurally conserved across the PRMT family, the selectivity of PARPi toward PRMT5 relative to other PRMTs will require further biochemical characterization. Recent studies on MTA-cooperative PRMT5 inhibitors have shown that selective engagement of PRMT5 within this conserved methyl-transferase architecture is achievable, but only after rigorous optimization and counter-screening for family selectivity [21,22]. Therefore, future work should examine whether olaparib or niraparib affects the enzymatic activity and canonical substrate methylation of additional PRMT family members.

The classic pharmacological mechanism of PARPi is the formation and trapping of a PARP-DNA complex at the site of single-strand damage, preventing DNA repair, leading to the accumulation of lethal double-strand break [23,24], which is the basis of the synthetic lethality of PARPi and HR repair deficiency. Therefore, PARPi is approved for the cancers with DNA repair deficiency, such as BRCA1/2-mutant breast, prostate, and pancreatic cancers [25,26,27]. Independent studies have shown that pharmacologic PRMT5 inhibition increases DNA damage and improves the antitumor activity of PARPi in breast and ovarian cancer models [28], further supporting the biological rationale for the synergistic effects observed here with olaparib plus EPZ015666. However, clinicians observed that PARPi is also effective in certain cancers that lack HR deficiency. For example, in patients with ovarian cancer without germline BRCA1/2 mutations, both niraparib and olaparib have prolonged progression-free survival [29,30]. Recently, Zeng reported that MTAP-deficient triple-negative breast cancer is susceptible to PARPi since PARPi down-regulates MAT2A by mediating METTL16 phosphorylation [12]. Although MAT2A was demonstrated not affected in non-breast cancer cells by PARPi in this study, it suggests that MTAP deficiency may enhance the cellular sensitivity to PARPi treatment. From a translational perspective, our data are also consistent with the recent emergence of MTA-cooperative PRMT5 inhibitors, such as MRTX1719 and AMG 193, which have shown selective antitumor activity in MTAP-deleted cancers in preclinical models and early clinical studies [21,22]. These advances suggest that MTAP deletion is becoming an increasingly actionable biomarker, and they position PARPi-based combinations as a potentially practical means to exploit the same vulnerability using agents that are already clinically available [31]. Recently, Zeng reported that MTAP-deficient triple-negative breast cancer is susceptible to PARPi since PARPi down-regulates MAT2A by mediating METTL16 phosphorylation [12]. In this study, we reveal that PARPi has a direct inhibitory effect on PRMT5 activity, which constitutes a synthetic lethality for MTAP-deficient tumors, enhancing DNA damage and the subsequent apoptosis.

In addition, the 76 overlapping differentially expressed genes identified after PARPi and PRMT5i treatment are best interpreted as evidence of pathway-level convergence, rather than as proof that individual genes are causal mediators of the observed phenotype. This interpretation is consistent with previous studies showing that PRMT5 inhibition downregulates DNA damage repair and DNA replication programs and can enhance response to PARP inhibition [19,28]. Although additional knockdown or rescue experiments for selected candidate genes would be valuable in future work, the positive ZIP scores observed in our combination analyses support a synergistic interaction under the analytical framework used in this study [14].

More broadly, tumor heterogeneity, lineage-specific biology, and differences in DNA repair capacity may influence the magnitude of response to this strategy across broader clinical settings. Thus, although our data support the existence of this vulnerability in the models examined here, broader validation across additional tumor types and molecular backgrounds will be important in future studies. In addition, potential toxicity in normal tissues remains an important translational consideration. In this regard, recent studies of MTA-cooperative PRMT5 inhibitors support the existence of a therapeutic window in MTAP-deleted tumors while emphasizing the need for careful evaluation in MTAP-wild-type contexts [21,22,31].

## 5. Conclusions

In conclusion, PARP inhibitors inactivate PRMT5 in vitro and in vivo and thereby enhance the vulnerability of MTAP-deficient tumors to PARP inhibition. MTAP-deficient tumors showed increased sensitivity to olaparib, and combination treatment with either MTDIA or EPZ015666 further improved antitumor efficacy. These findings support PARP inhibitor-based strategies as a promising therapeutic approach for MTAP-deficient cancers and provide a rationale for targeting the MTAP–PRMT5 axis in combination therapy.

## Data Availability

All the data and figures are available following the author’s permission. The RNA-seq data was deposited to Sequence Read Archive (SRA) under accession number PRJNA1336408.

## Supplementary Materials

The following supporting information can be downloaded at: https://www.mdpi.com/article/10.3390/cancers18091335/s1, Figure S1: The mRNA levels of MAT2A following PARPi treatment in JF-305 cells; Figure S2: Restoring MTAP expression in A549 cells; Figure S3: Original full-length WB images corresponding to Figure 1; Figure S4: Original full-length WB images corresponding to Figure 2; Figure S5: Original full-length WB images corresponding to Figure 3; Figure S6: Original full-length WB images corresponding to Supplementary Figure S2; Table S1: PRMT5-Olaparib interaction; Table S2: PRMT5-Niraparib interaction; Table S3: Reagents, kits and material information; Table S4: Oligo nucleotides used in the study.
