# Supplementary material for: Inactivation of PRMT5 by PARP Inhibitors Confers High Susceptibility in MTAP-Deficient Cancers
Source: Cancers (Basel). 2026 Apr 22;18(9):1335. doi: 10.3390/cancers18091335 (PMC13163060; doi:10.3390/cancers18091335)
Supplement: Supplementary file 1 [file cancers-18-01335-s001.zip › HCT116 cells STR report.pdf]

HCT 116 cells STR report

Method: An appropriate amount of "HCT 116" cells (Cell number PC-H2024080820,1×10^6) were used TIANamp Genomic DNA Kit to extract DNA, 20 STR loci and gender identification loci were amplified by Microreader™21 ID System, PCR product detection was performed by GenReader 7010 genetic analyzer, detection results were analyzed by GeneMapper Software6 (Applied Biosystems), and compared with ExPASy databases.

Experimental result:

1. The results of negative and positive control were correct.
2. The genotyping results of STR locus of "HCT 116" cell line is shown in the following table.

Conclusion:

1. The genomic DNA of "HCT 116" cell line is clear and the result of genotyping is good.
2. The results of STR typing showed that no cross contamination of human cell was found in the cell line of "HCT 116" cell line.
3. The DNA typing of the cell line was 91.43% matched with the cell type in the cell bank, and the cell line name was HCT 116.

Appendix I: The genotyping results of STR locus of "HCT 116" cell line.

STR Loci	Sample: PC-H2024080820	Database：HCT 116
Amelogenin	X	X
CSF1PO	7,10	7,9,10,11
D2S1338	16	16
D3S1358	12,17,18,19	12,18,19
D5S818	10,11	10,11
D7S820	11,12	11,12
D8S1179	12,14	12,14
D13S317	10,12	10,12
D16S539	11,13	11,12,13,14
D18S51	16,17	16,17
D19S433	12,13	12
D21S11	29,30	29,30
FGA	18,23	18,23
PentaD	9,13	9,13
PentaE	13,14	13,14
TH01	8,9	8,9
TPOX	8	8
vWA	17,22	17,22
D6S1043	13	
D12S391	17,21	
D2S441	11,12	
The ExPASy database has a matching rate of 91.43%，The number of matched bits is 17 （https://www.cellosaurus.org/index.html）		

Note:

1. According to the cell STR identification standard established by the International Cell Line Authentication Committee (ICLAC), when the matching degree of cell lines is ≥80%, they are considered to be correlated, that is, derived from common ancestral cells; The matching degree is between 55% and 80%, and the correlation needs to be further verified. Less than 55% indicates no correlation between the two.
2. The effective peak of the map was the real PCR band; Small peaks and nonspecific bands were ignored in the calculation.
3. The default ExPASy STR data comparing the results, the data sources include ATCC, DSMZ, JCRB cell library and documents and records, such as https://www.cellosaurus.org/index.html database entry.

Appendix II: The genotyping results of STR locus of "HCT 116" (Cell number PC-H2024080820) cell line.

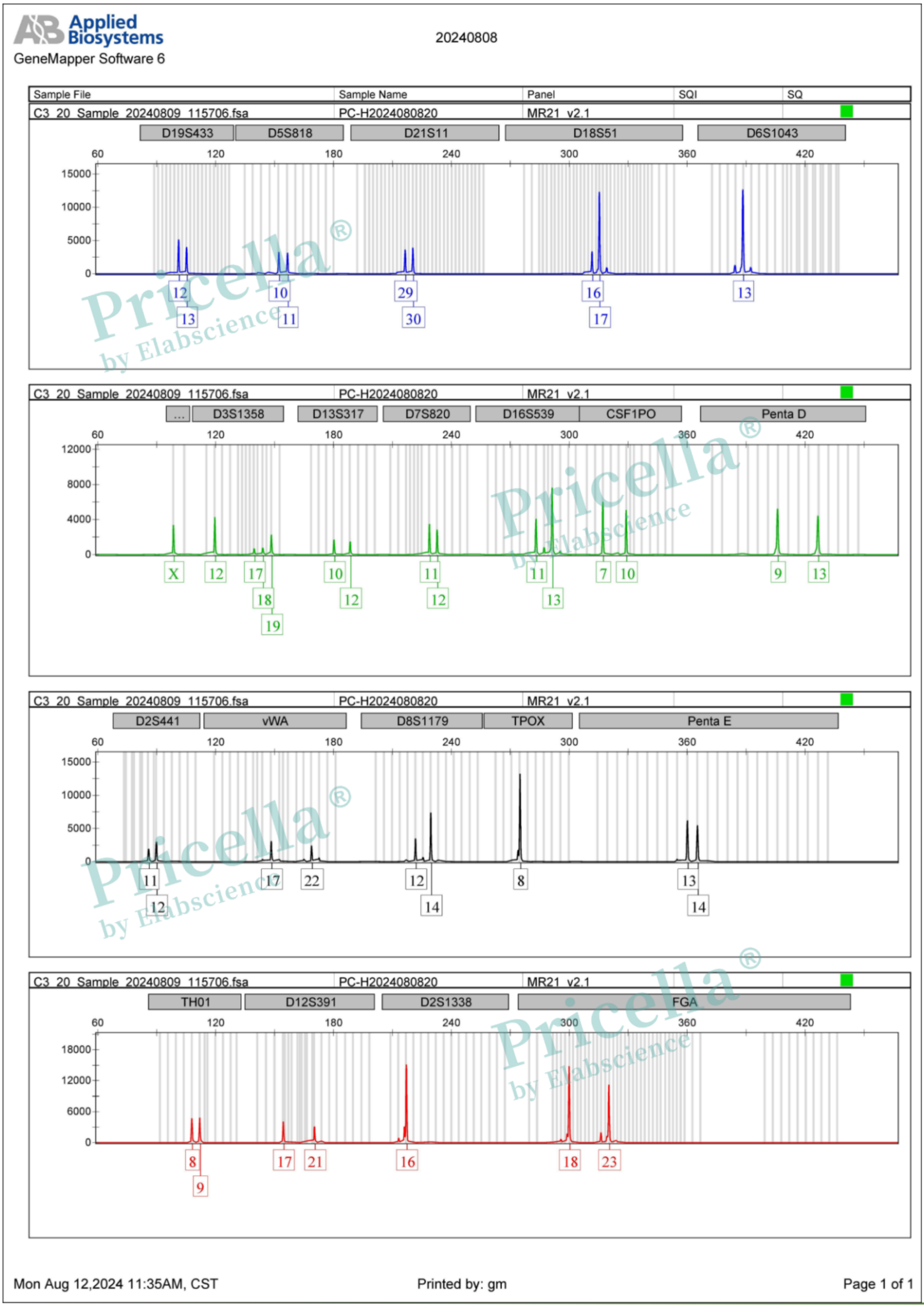

Web: www.procell.com.cn
Tel: 400-999-2100
Email: techsupport@procell.com.cn
Add: Building C4,3rd Phase of Biomedical Park, #858 Gaoxin Road, Donghu Hi-Tech Development
