# Supplementary material for: Inactivation of PRMT5 by PARP Inhibitors Confers High Susceptibility in MTAP-Deficient Cancers
Source: Cancers (Basel). 2026 Apr 22;18(9):1335. doi: 10.3390/cancers18091335 (PMC13163060; doi:10.3390/cancers18091335)
Supplement: Supplementary file 1 [file cancers-18-01335-s001.zip › HEK-293T cells STR report.pdf]

293T [HEK-293T] cells STR report

Method: An appropriate amount of "293T [HEK-293T]" cells (Cell number PC-H2024091003,1×10^6) were used TIANamp Genomic DNA Kit to extract DNA, 20 STR loci and gender identification loci were amplified by Microreader™21 ID System, PCR product detection was performed by GenReader 7010 genetic analyzer, detection results were analyzed by GeneMapper Software6 (Applied Biosystems), and compared with ExPASy databases.

Experimental result:

1. The results of negative and positive control were correct.
2. The genotyping results of STR locus of "293T [HEK-293T]" cell line is shown in the following table.

Conclusion:

1. The genomic DNA of "293T [HEK-293T]" cell line is clear and the result of genotyping is good.
2. The results of STR typing showed that no cross contamination of human cell was found in the cell line of "293T [HEK-293T]" cell line.
3. The DNA typing of the cell line was 98.55% matched with the cell type in the cell bank, and the cell line name was HEK293T.

Appendix I: The genotyping results of STR locus of "293T [HEK-293T]" cell line.

STR Loci	Sample: PC-H2024091003	Database: HEK293T
Amelogenin	X	X
CSF1PO	11,12	11,12
D2S1338	19	19
D3S1358	15,16,17	15,16,17
D5S818	8,9	8,9
D7S820	11	11
D8S1179	11,12,14	12,14
D13S317	12,14	12,14
D16S539	9,13	9,13
D18S51	17,18	17,18
D19S433	18	18
D21S11	28,30.2	28,30.2
FGA	23	23
PentaD	9,10	9,10
PentaE	7,15	7,15
TH01	7,9.3	7,9.3
TPOX	11	11
vWA	16,19	16,19
D6S1043	11	
D12S391	19,21	19,21
D2S441	11,15	11,15
The ExPASy database has a matching rate of 98.55%，The number of matched bits is 19（https://www.cellosaurus.org/index.html）		

Note:

1. According to the cell STR identification standard established by the International Cell Line Authentication Committee (ICLAC), when the matching degree of cell lines is ≥80%, they are considered to be correlated, that is, derived from common ancestral cells; The matching degree is between 55% and 80%, and the correlation needs to be further verified. Less than 55% indicates no correlation between the two.
2. The effective peak of the map was the real PCR band; Small peaks and nonspecific bands were ignored in the calculation.
3. The default ExPASy STR data comparing the results, the data sources include ATCC, DSMZ, JCRB cell library and documents and records, such as https://www.cellosaurus.org/index.html database entry.

Appendix II: The genotyping results of STR locus of "293T [HEK-293T]" (Cell number PC-H2024091003) cell line.

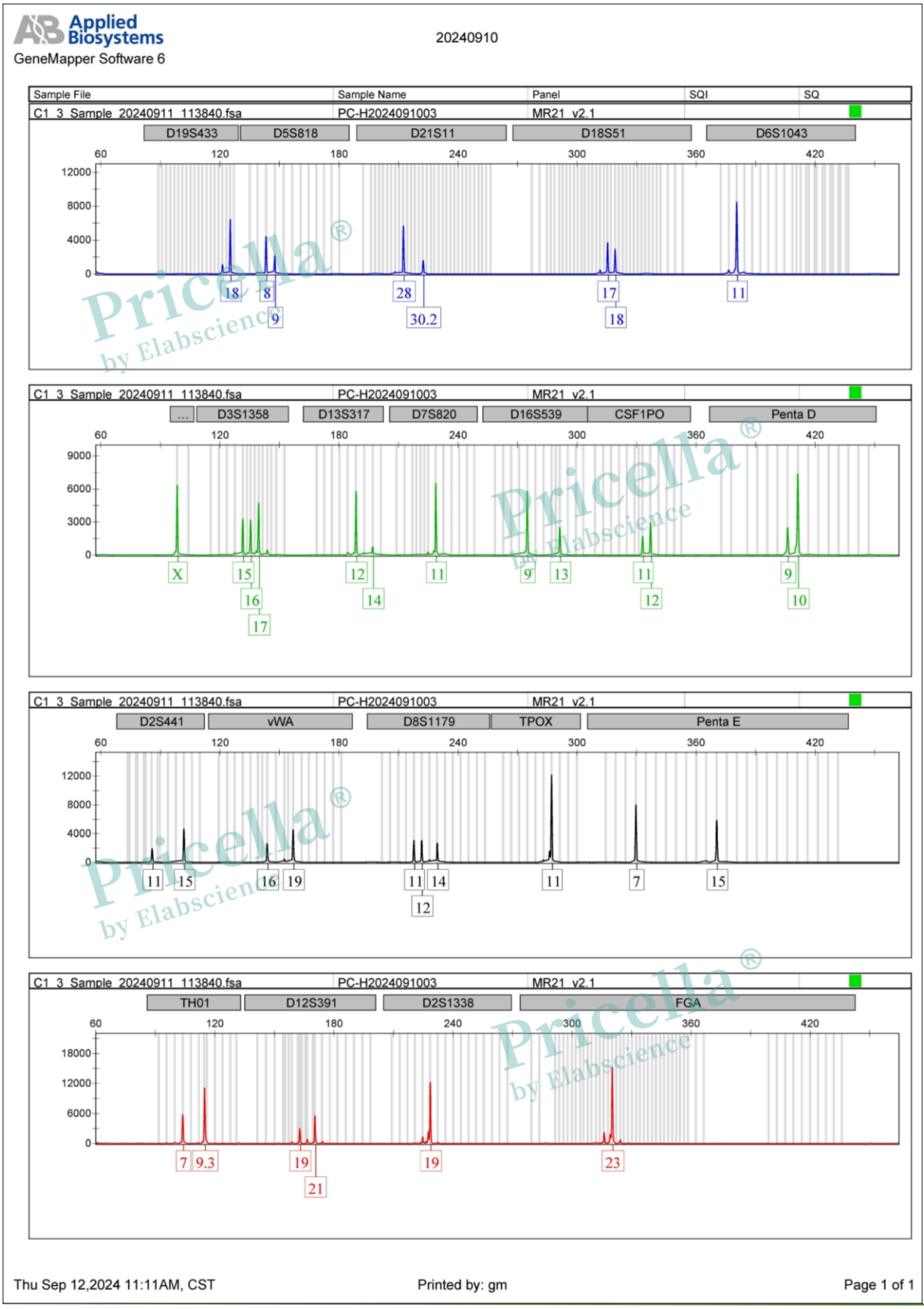

Web: www.procell.com.cn
Tel: 400-999-2100
Email: techsupport@procell.com.cn
Add: Building C4,3rd Phase of Biomedical Park, #858 Gaoxin Road, Donghu Hi-Tech Development
