# Supplementary material for: Inactivation of PRMT5 by PARP Inhibitors Confers High Susceptibility in MTAP-Deficient Cancers
Source: Cancers (Basel). 2026 Apr 22;18(9):1335. doi: 10.3390/cancers18091335 (PMC13163060; doi:10.3390/cancers18091335)

Applied Biosystems

GeneMapper 4.0

CellLineAuthentication

Sample File	Sample Name	Panel	SQI	OS	SQ
09_A02_CellLineAuthentication-0108.fsa	JF-305	STR Profile 1-human			

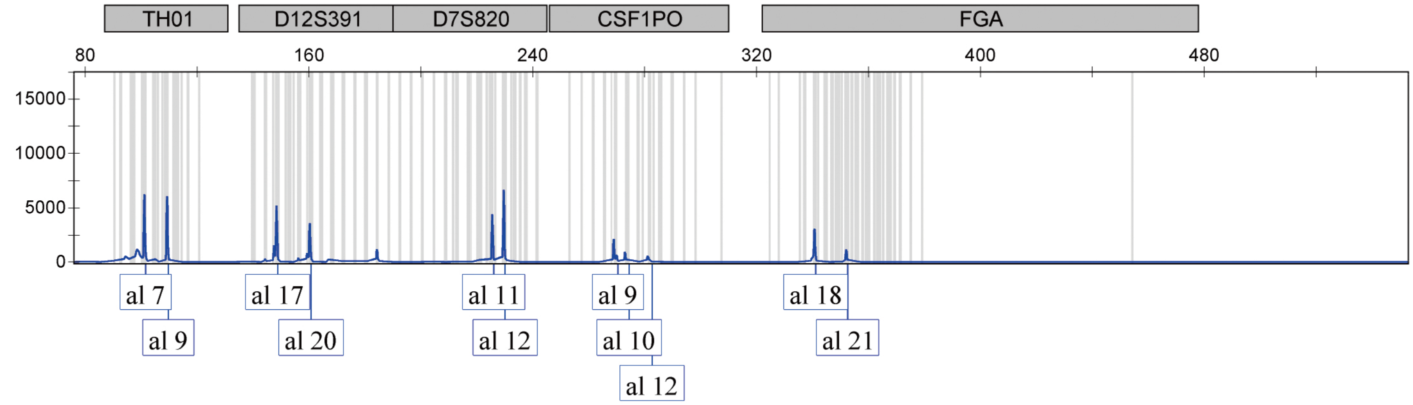

Sample File	Sample Name	Panel	SQI	OS	SQ
09_A02_CellLineAuthentication-0108.fsa	JF-305	STR Profile 1-human			

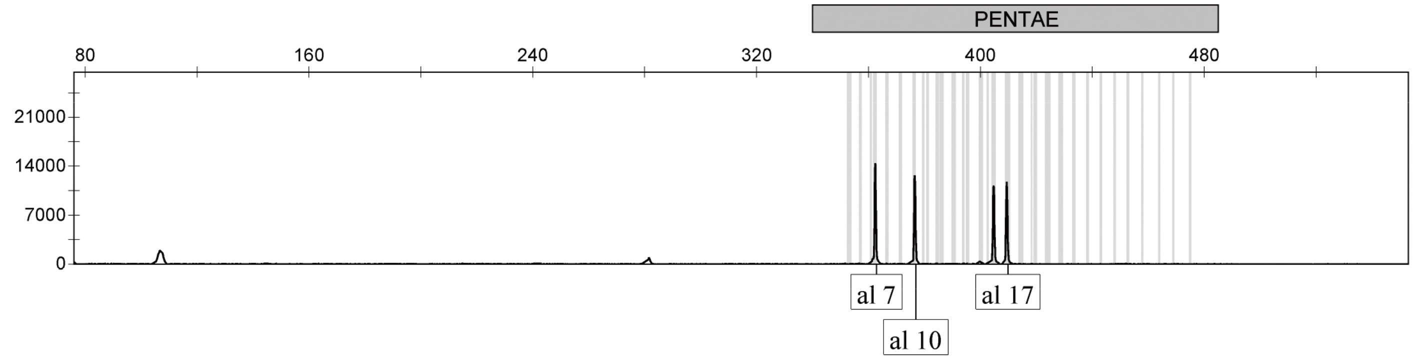

Sample File	Sample Name	Panel	SQI	OS	SQ
41_A06_CellLineAuthentication-0108.fsa	JF-305	STR Profile 2-human			

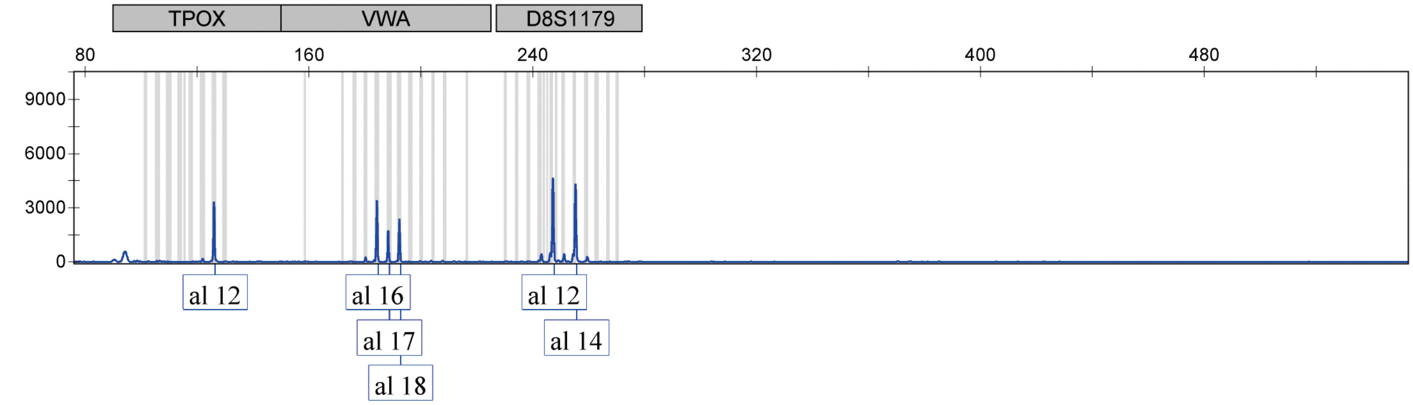

Sample File	Sample Name	Panel	SQI	OS	SQ
41_A06_CellLineAuthentication-0108.fsa	JF-305	STR Profile 2-human			

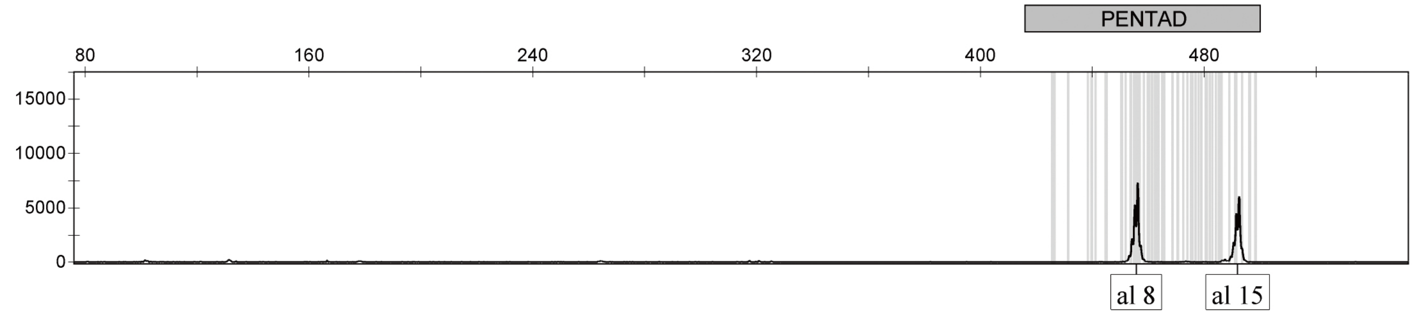

Sample File	Sample Name	Panel	SQI	OS	SQ
65_A09_CellLineAuthentication-0108.fsa	JF-305	STR Profile 3-human			

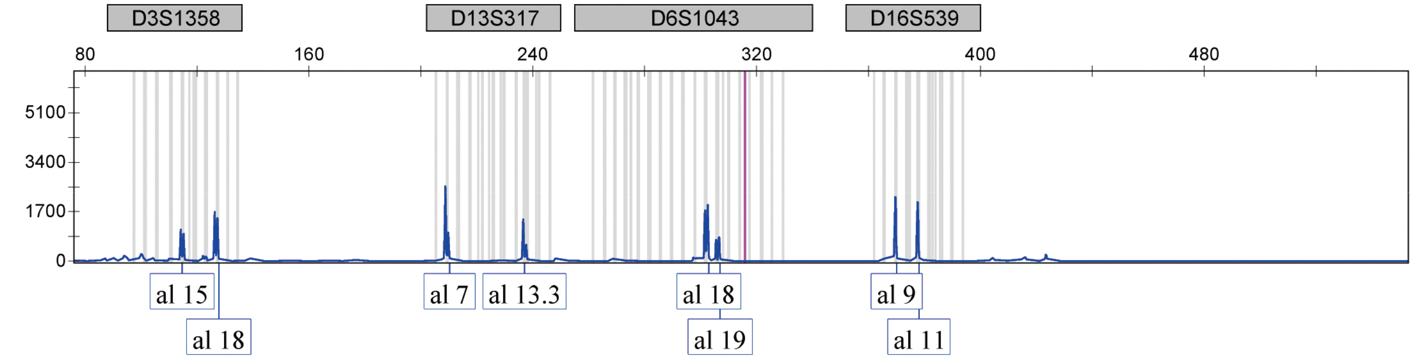

Sample File	Sample Name	Panel	SQI	OS	SQ
65_A09_CellLineAuthentication-0108.fsa	JF-305	STR Profile 3-human			

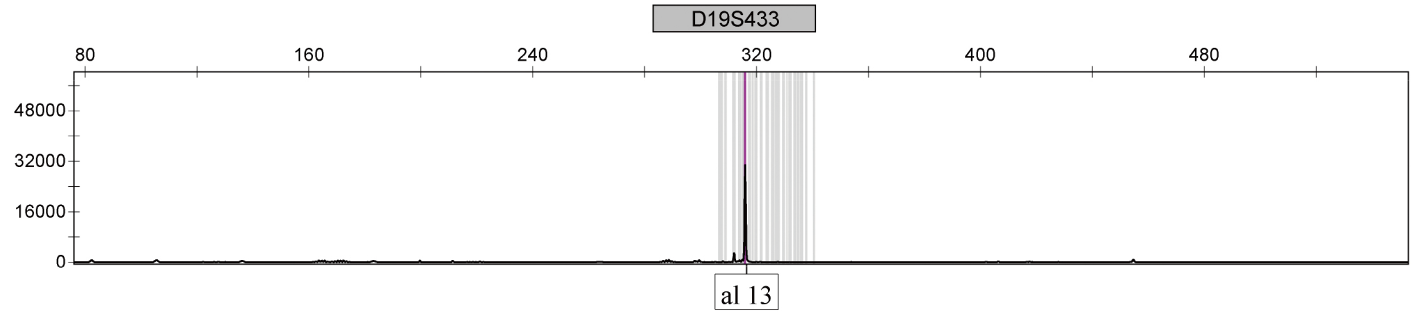

Sample File	Sample Name	Panel	SQI	OS	SQ
89_A12_CellLineAuthentication-0108.fsa	JF-305	STR Profile 4-human			

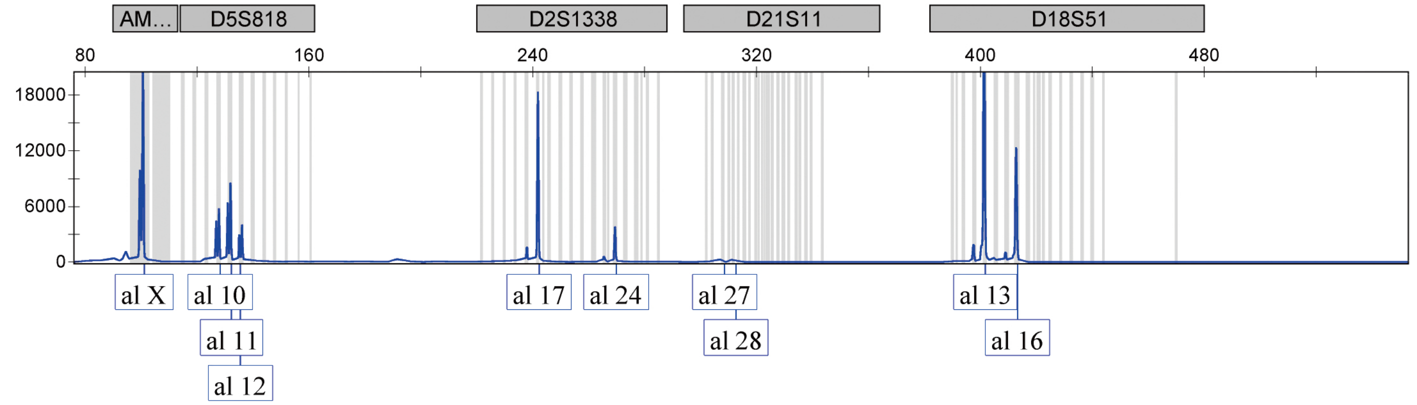

89_A12_CellLineAuthentication-0108.fsa	JF-305	STR Profile 4-human			

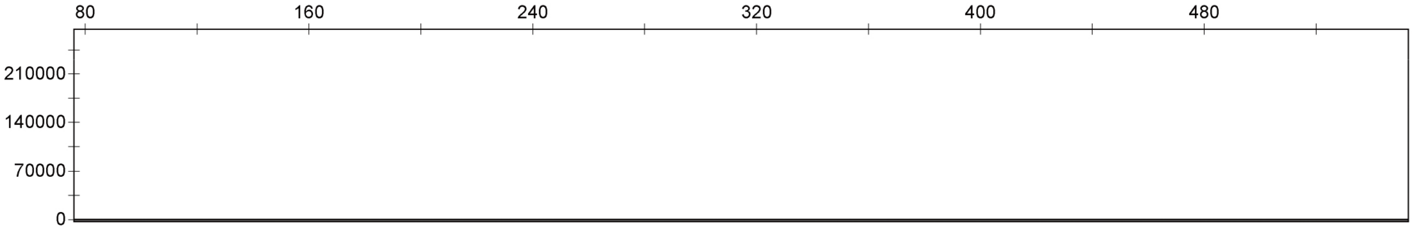

Supplement: Supplementary file 1 [file cancers-18-01335-s001.zip › JF-305 cells STR report.pdf]
