# Supplementary material for: Inactivation of PRMT5 by PARP Inhibitors Confers High Susceptibility in MTAP-Deficient Cancers
Source: Cancers (Basel). 2026 Apr 22;18(9):1335. doi: 10.3390/cancers18091335 (PMC13163060; doi:10.3390/cancers18091335)
Supplement: Supplementary file 1 [file cancers-18-01335-s001.zip › Laboratory Animal Ethics Review Resolution of SUSTech.pdf]

南方科技大学实验动物伦理审查决议书

Laboratory Animal Ethics Review Resolution of SUSTech

决议编号<br>Resolution number	SUSTech-JY2020142
实验动物<br>Laboratory animal	小鼠
课题名称<br>**Program name**	MTAP-PRMT5-WDR5 信号通路调控肿瘤放化疗敏感性的分子机制及其肿瘤学意义
动物实验项目名称<br>**Animal protocol name**	大肠癌裸鼠成瘤及药物试验
申请人<br>**Applicant**	杜长征
受理编号<br>Acceptance number	SUSTech-2020-157
申请时间<br>Application time	2020 年 7 月 24 日
是否通过初审<br>Whether to pass the preliminary review	☑通过 **Agree**<br>☐不通过 **Disagree**
会议或通讯审查的时间<br>Time of meeting or newsletter review	2020 年 7 月 27 日
审查决议<br>Review resolution	☑可以进行实验 **Approved**<br>☐调整方案后，可以进行实验 **To be revised**<br>☐ 不同意 **Disapproved**
主任或授权的副主任签名<br>Signature of the Chairman or authorized deputy Chairman	[signature] 日期 Date 2020年 7月 3 1日
