# Supplementary material for: Inactivation of PRMT5 by PARP Inhibitors Confers High Susceptibility in MTAP-Deficient Cancers
Source: Cancers (Basel). 2026 Apr 22;18(9):1335. doi: 10.3390/cancers18091335 (PMC13163060; doi:10.3390/cancers18091335)
Supplement: Supplementary file 1 [file cancers-18-01335-s001.zip › Table S1-S2.pdf]

Table S1. PRMT5-Olaparib interaction.

Hydrogen bonds					
**Index**	**Residue**	**AA**	**Distance (Å)**	**Ligand Atom**	**Protein Atom**
1	365	GLY	3.27	6163-O	3476-N
2	444	GLU	2.81	6166-F	4237-OE1
**Hydrophobic interactions**					
**Index**	**Residue**	**AA**	**Distance (Å)**	**Ligand Atom**	**Protein Atom**
1	314	PRO	3.7	6139-C	2965-C
2	315	LEU	2.77	6139-C	2976-CD1
3	363	VAL	3.83	6139-C	3464-CG1
4	370	PRO	2.88	6163-O	3519-CG
5	371	LEU	3.13	6139-C	3526-CG
6	420	MET	2.94	6139-C	4013-CE
7	436	LEU	3.39	6166-F	4162-CA

Table S2. PRMT5-Niraparib interaction.

Hydrogen bonds					
**Index**	**Residue**	**AA**	**Distance (Å)**	**Ligand Atom**	**Protein Atom**
1	328	GLU	3	6135-N	3108-OE2
2	393	LYS	3.08	6158-O	3744-N
**Hydrophobic interactions**					
**Index**	**Residue**	**AA**	**Distance (Å)**	**Ligand Atom**	**Protein Atom**
1	314	PRO	3.77	6139-C	2965-C
2	315	LEU	3.41	6139-C	2971-CA
3	327	PHE	3.35	6139-C	3092-CB
4	370	PRO	2.91	6135-N	3519-CG
5	420	MET	3.4	6135-N	4013-CE
6	436	LEU	3.74	6135-N	4168-CD2
