# Supplementary material for: Inactivation of PRMT5 by PARP Inhibitors Confers High Susceptibility in MTAP-Deficient Cancers
Source: Cancers (Basel). 2026 Apr 22;18(9):1335. doi: 10.3390/cancers18091335 (PMC13163060; doi:10.3390/cancers18091335)
Supplement: Supplementary file 1 [file cancers-18-01335-s001.zip › Table S3.pdf]

Table S3. Reagents, kits and material information.

REAGENT or RESOURCE	SOURCE	IDENTIFIER
**Antibodies**		
MTAP Polyclonal antibody	Proteintech	Cat#111475-1-AP
β-Actin (8H10D10) Mouse mAb	CST	Cat#3700S
PRMT5 (D5P2T) Rabbit mAb	CST	Cat#79998S
Rabbit polyclonal to Histone H4 (symmetric di methyl R3)	Abcam	Ab5823
Mouse monoclonal [9F3] to gamma H2A.X (phospho S139)	Abcam	Ab26350
Histone H4 (L64C1) Mouse mAb	CST	Cat#2935T
Alexa Fluor® 594 Conjugated Goat anti-mouse IgG Goat Polyclonal Antibody	HUABIO	Cat#HA1112
Peroxidase-Conjugated Goat anti-Rabbit IgG (H+L)	ZSGB-BIO	Cat#ZB-2301
Peroxidase-Conjugated Goat anti-Mouse IgG (H+L)	ZSGB-BIO	Cat#ZB-2305
**Chemicals and reagents**		
DMEM	Gibco	Cat#11995065
RPMI 1640 medium	Gibco	Cat#61870036
Penicillin/streptomycin	Gibco	Cat#15140122
Fetal bovine serum	Gibco	Cat#10099141C
PBS	Biosharp	Cat#BL302A
Puromycin	MedChemExpress	Cat#HY-K1057
Lipofectamine™ 2000	Invitrogen	Cat#11668030
paraformaldehyde	Sigma-Aldrich	Cat#158127
0.25% EDTA-Trypsin	Gibco	Cat#25200072
Trypan blue stain	Gibco	Cat#15250061
Matrigel Matrix	Corning	Cat#2363002
TRIzol™	Invitrogen	Cat#15596026
ECL	Advansta	Cat#K-12045-D50
Hot Start Takara Taq DNA Polymerase	Takara	Cat#R007A
Crystal violet	Macklin	Cat#548-62-9
Cell Counting Kit-8	Beyotime	C0040
Beyo3D™ DAPI	Beyotime	C1341S
Thermo Scientific Triton X-100 Surfact-Amps	ThermoFisher	Cat#85111
Recombinant H4 protein	Abcam	Ab198115
Recombinant PRMT5 protein	Active Motif	Cat#31393
Olaparib	MCE	Cat#HY-10162
Niraparib	MCE	Cat#HY-10619
EPZ015666	MCE	Cat#HY-12727
MTDIA	MCE	Cat#HY-101496

DMSO	ThermoFisher	
**Cell Lines**		
HEK-293T	ATCC	Cat#CRL-3216
JF-305	Prof. Jing Gao	
HCT116	ATCC	Cat#CCL-247EMT
HT-29	ATCC	Cat#HTB-38
A549	ATCC	Cat#CCL-185
**Plasmids**		
lentiCRISPR v2	Addgene	Cat#52961
pLV [Exp]-EGFP/Puro-EF1A>mCherry	VectorBuilder, China	Ecoli(VB010000-9494mhq)
pLKO.1-TRC cloning vector	Addgene	Cat#10878
pLKO.1-blast	Addgene	Cat#26655
psPAX2	Addgene	Cat#12260
pMD2.G	Addgene	Cat#12259
pLVX-M-puro	Addgene	Cat#125839
pcDNA3.1-3×Flag	Addgene	Cat#182494
**Critical Commercial Kits**		
DAB Substrate Kit	CST	Cat#8059
NEBNext Ultra II Directional RNA Library Prep Kit for Illumina	NEB	Cat#E7760
FastPure Cell/T issue Total RNA isolution Kit V2	Vazyme	Cat#RC112-01
HiScript III 1st Strand cDNA Synthesis Kit (+gDNA wiper)	Vazyme	Cat#R312-02
TransStart Top Green qPCR SuperMix	TransGen Biotech	Cat#AQ132-21
Hieff Clone® Zero TOPO-TA Cloning Kit	Yeasen	Cat#10906ES20
QIAprep Spin Miniprep Kit	Qiagen	Cat#27104
Pierce BCA Protein Assay Kit	ThermoFisher	Cat#23227
Protease inhibitor cocktail	Beyotime	P1005
