# Supplementary material for: Inactivation of PRMT5 by PARP Inhibitors Confers High Susceptibility in MTAP-Deficient Cancers
Source: Cancers (Basel). 2026 Apr 22;18(9):1335. doi: 10.3390/cancers18091335 (PMC13163060; doi:10.3390/cancers18091335)
Supplement: Supplementary file 1 [file cancers-18-01335-s001.zip › Table S4.pdf]

Table S4. Oligo nucleotides used in the study sgRNA sequences.

Name	F or R	Sequence（5' to 3'）
sgMTAP #1	F	CACCGTCATCTCACCTTCACGGCGG
	R	AAACCCGCCGTGAAGGTGAGATGAC
sgMTAP #2	F	CACCGTCTGCCCGGGAGCTAAAACG
	R	AAACCGTTTTAGCTCCCGGGCAGAC

Primers for constructing MTAP overexpression plasmid

Name	F or R	Sequence（5' to 3'）
MTAP	F	CGGGATCCATGCGCCCGGCCCGT
	R	CCGGAATTCTTAATGTCTTGGTAATAAAAC

shRNA sequences

Name	F or R	Sequence（5' to 3'）
shCtrl	F	CCGGCCTAAGGTTAAGTCGCCCTCGCTCGAGCGAGG GCGACTTAACCTTAGGTTTTTG
	R	AATTCAAAAACCTAAGGTTAAGTCGCCCTCGCTCGAG CGAGGGCGACTTAACCTTAGG
shPRMT5 #1	F	CCGGCCTCAAGAACTCCCTGGAATACTCGAGTATTCC AGGGAGTTCTTGAGGTTTTTG
	R	AATTCAAAAACCTCAAGAACTCCCTGGAATACTCGA GTATTCCAGGGAGTTCTTGAGGCCGG
shPRMT5 #2	F	CCGGGCCCAGTTTGAGATGCCTTATCTCGAGATAAGG CATCTCAAACTGGGCTTTTTG
	R	AATTCAAAAAGCCCAGTTTGAGATGCCTTATCTCGAG ATAAGGCATCTCAAACTGGGCCCGG

Primers for RT-qPCR

Name	F or R	Sequence（5' to 3'）
B2M	F	AGGCTATCCAGCGTACTCCA
	R	CTGCTTACATGTCTCGATCCCA
MTAP	F	TCTTGTGCCAGAGGAGTGTG
	R	ACCGAAACTGCTTCCTCGTG
PRMT5	F	ACTAGTCATCCCGGAGAAGCA
	R	GCGTATTCCAGTCCCTTCCT
MAT2A	F	TTTGCTAACCTGACGCCCTT
	R	TCCAGCTTCTCCGACTCTTTC
