# Supplementary figures and images for: Inactivation of PRMT5 by PARP Inhibitors Confers High Susceptibility in MTAP-Deficient Cancers
Source: Cancers (Basel). 2026 Apr 22;18(9):1335. doi: 10.3390/cancers18091335 (PMC13163060; doi:10.3390/cancers18091335)

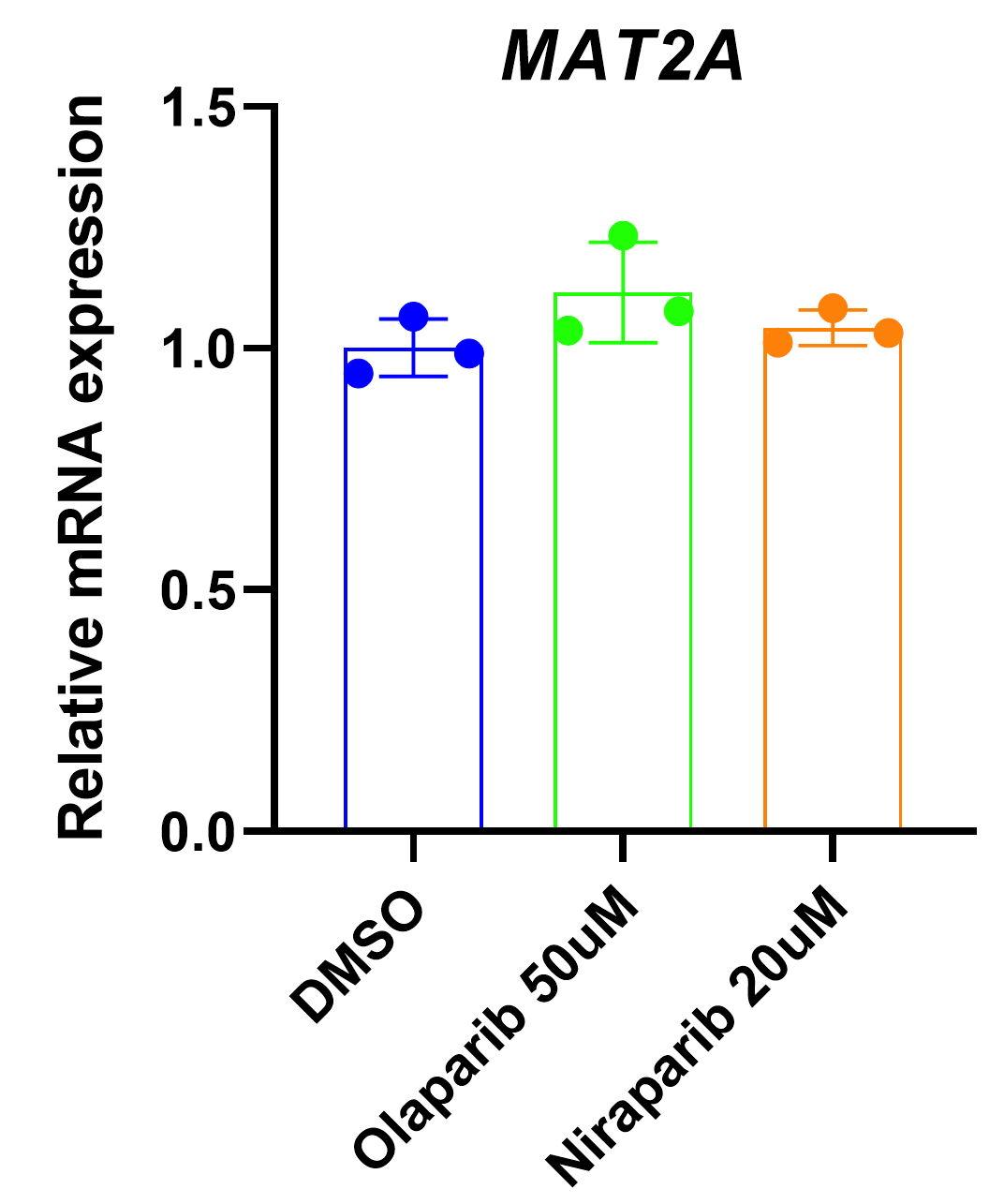

Supplement: Supplementary file 1 [file cancers-18-01335-s001.zip › Figure S1. The mRNA levels of MAT2A following PARPi treatment in JF-305 cells.tif]

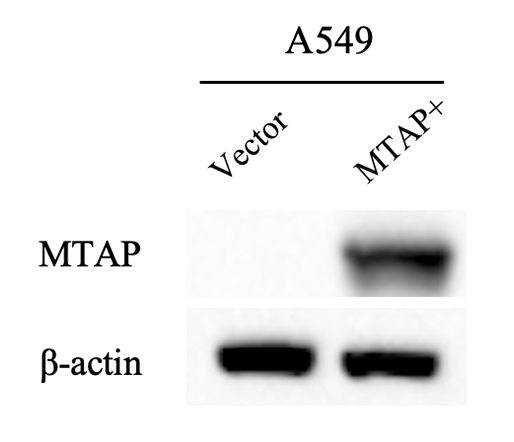

Supplement: Supplementary file 1 [file cancers-18-01335-s001.zip › Figure S2. Restoring MTAP expression in A549 cells.tif]

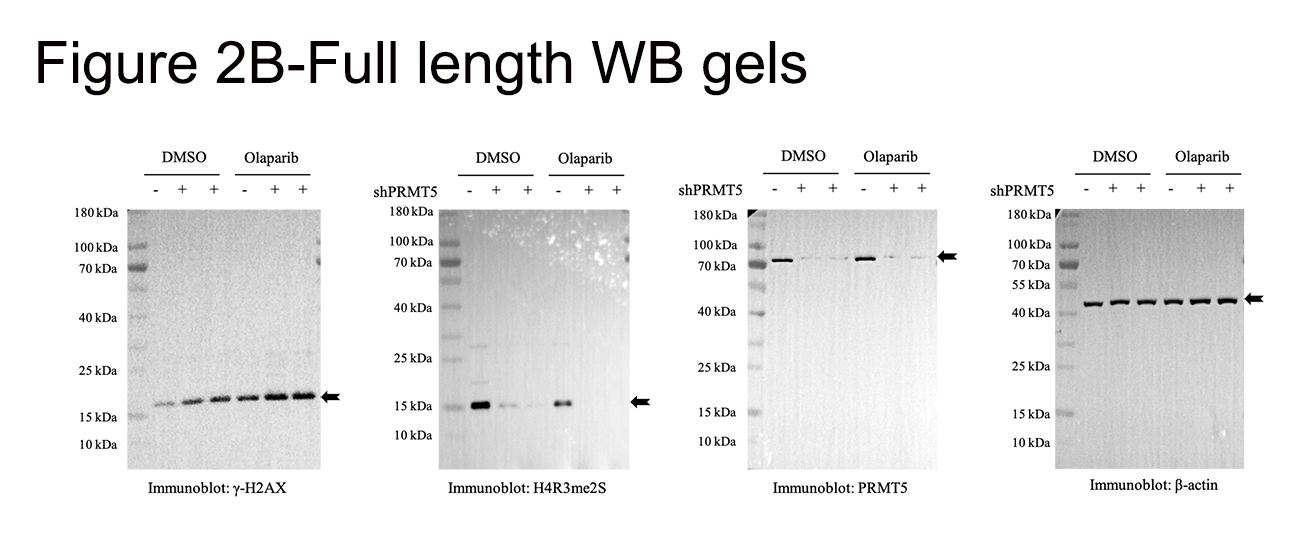

Supplement: Supplementary file 1 [file cancers-18-01335-s001.zip › Figure S4. Original full-length WB images corresponding to Figure 2.tif]

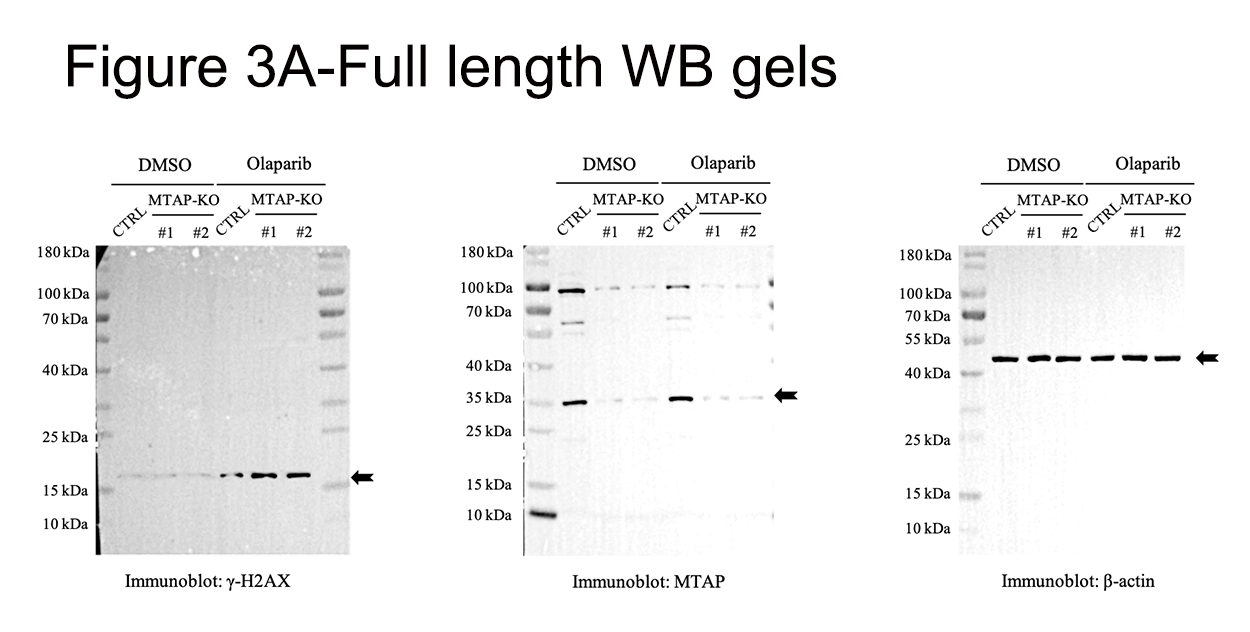

Supplement: Supplementary file 1 [file cancers-18-01335-s001.zip › Figure S5. Original full-length WB images corresponding to Figure 3.tif]

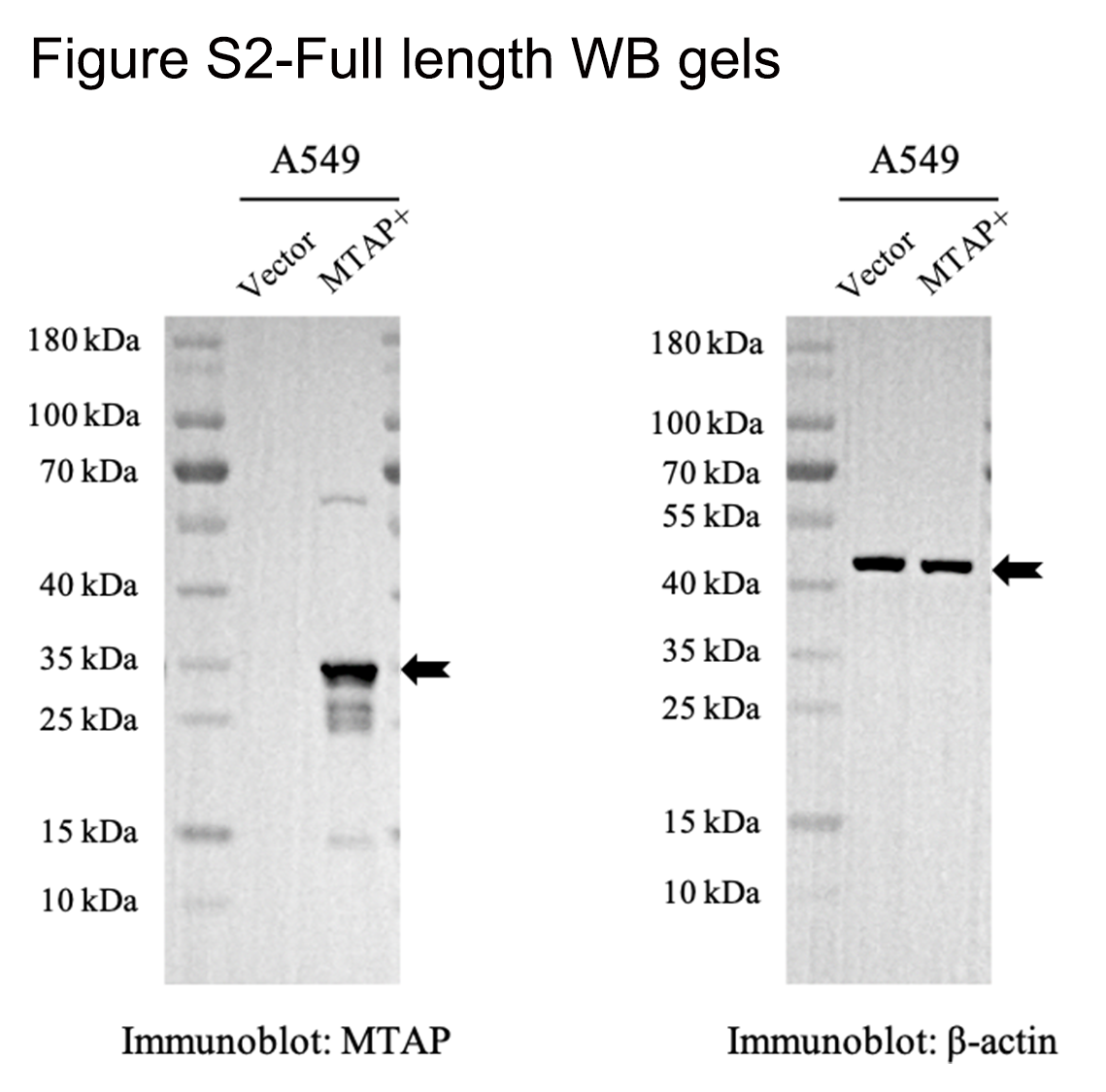

Supplement: Supplementary file 1 [file cancers-18-01335-s001.zip › Figure S6. Original full-length WB images corresponding to Supplementary Figure S2.tif]
